# Human Defensins: Structure, Function, and Potential as Therapeutic Antimicrobial Agents with Highlights Against SARS CoV-2

**DOI:** 10.1007/s12602-024-10436-8

**Published:** 2024-12-18

**Authors:** Maryam Nagib, Ahmed M. Sayed, Ahmed H. Korany, Karim Abdelkader, Falah H. Shari, William G. Mackay, Mostafa E. Rateb

**Affiliations:** 1 School of Computing Engineering and Physical Sciences, University of the West of Scotland, Paisley, Scotland PA12BE UK; 2 Department of Pharmacognosy, College of Pharmacy, Almaaqal University, Basrah, 61014 Iraq; 3 Department of Microbiology and Immunology, Faculty of Pharmacy, Nahda University, Beni Suef, 62513 Egypt; 4 Department of Microbiology and Immunology, Faculty of Pharmacy, Beni-Suef University, Beni Suef, 62511 Egypt; 5 Department of Clinical Biochemistry, College of Pharmacy, Almaaqal University, Basrah, 61014 Iraq; 6 School of Health and Life Sciences, University of the West of Scotland, Blantyre, Glasgow G72 0LH UK

**Keywords:** Human defensins, Antimicrobial peptides, SARS CoV-2, In silico analysis

## Abstract

The human defensins are a group of cationic antimicrobial peptides that range in size from 2 to 5 kDa and share a common structural motif of six disulphide-linked cysteines. Several naturally occurring human α- and β-defensins have been identified over the past two decades. They have a wide variety of antimicrobial effects, and their potential to avoid the development of resistance to antimicrobial treatment makes them attractive as therapeutic agents. Human defensins have recently been the focus of medical and molecular biology studies due to their promising application in medicine and the pharmaceutical industry. This work aims to provide a comprehensive summary of the current developments of human defensins, including their identification, categorization, molecular features, expression, modes of action, and potential application in medical settings. Current obstacles and future opportunities for using human defensins are also covered. Furthermore, we shed light on the potential of this class as an antiviral agent, particularly against SARS CoV-2, by providing an in silico-based investigation of their plausible mechanisms of action.

**Supplementary Information:**

The online version contains supplementary material available at 10.1007/s12602-024-10436-8.

## Introduction

Antimicrobial peptides are natural short peptides with different physiochemical properties (cationic or amphipathic) that play a crucial role in protecting the human host from bacterial, viral, or fungal infections [1, 2].

Human Defensins (HDs) constitute a family of arginine-rich antimicrobial peptides that carry out multifaceted immunomodulatory functions within the innate immune system, responsible for multiple roles in various processes related to human host defence [3]. This group of antimicrobial peptides is phylogenetically ancient, as evidenced by their presence in lower organisms [4]. As such, defensins provide a key link between the innate immune system and the adaptive immune response in humans [4].

These host defence peptides are small at approximately 2–5 kDa in size and structurally characterised by a β-sheet core stabilised by three conserved intramolecular disulphide bonds [5]. According to the respective disulphide topology of defensins, there are three family subcategories: α, β, and θ [6]. Within humans, only α and β defensins exist, whilst θ defensins are exclusively found in *Rhesus macaques* [7], RNA transcripts homologous to those of the *Rhesus macaques* θ defensin gene are also found in humans [8]. The presence of a premature stop codon in the gene’s upstream signalling sequence prevents successful translation of the θ defensin in humans [8].

Alongside the antimicrobial activities of these peptides, HDs also play different roles in other immunomodulatory functions. Human α-defensins, for example, inhibit fibrinolysis by hindering the binding of plasminogen and fibrin, thereby modulating thrombotic pathology [9]. Additionally, HDs in circulating blood may adhere to the endothelium, contributing to inflammation in the vasculature under pathological conditions [9, 10]. This group of defensins is also partly responsible for the regulation of antiprotease secretion in the lungs at sites of inflammation through the release of secretory leukocyte protease inhibitors and elafin in the bronchial epithelium [11]. Further to this, human α-defensins also induce the catabolism of low-density lipoprotein (LDL) by vascular cells, indicating their ability to contribute to atherogenesis [12].

In tumorigenesis, HDs have been found to play contradictory roles [13]. Under different physiological conditions, HDs were found to either have a proliferative or suppressive effect on different carcinomas [14, 15]. The α-defensins HNP1-3 have been frequently detected in different cancer patients’ tumours and biological fluids [15]. This has been attributed to the presence of tumour-infiltrating neutrophils, although some studies have suggested that tumour cells themselves may be producing HNP1-3, though this remains mechanistically unclear [16]. Some studies infer that HNP1-3 promotes tumour cell proliferation [17]; conversely, however, more recent studies suggest that these defensins carry out various anti-tumour activities such as the inhibition of angiogenesis, induction of apoptosis, and altering the immune milieu through the recruitment of immature dendritic cells [18, 19].

As most cancers develop in epithelial cells and tissues where β-defensins are expressed, there has been considerable interest in identifying the functional role of defensins in carcinomas [20]. HBD1 was found to be downregulated in most carcinomas, with evidence suggesting its function as a tumour suppressor [21–23]. On the other hand, HBD3 was found to stimulate tumour growth and migration and further assist with the recruitment of tumour-associated macrophages, further promoting carcinoma progression [24–26]. Alternatively, HBD2 regulation seems to be only partially understood, with its role in influencing tumorigenesis varying among different cancers.

The importance of HDs in innate and adaptive immunity is particularly evident when observing the increased frequency of common infections in defensin-deficient patients [27]. While individual HDs have distinct antimicrobial activity profiles, there is considerable interest in investigating the synergistic uses of multiple defensin combinations.

All this to say, human defensins play complex roles in regulating the many immunomodulatory functions of the innate immune system, and their antithetical activities have earned them the title of a “double-edged sword” in host immunity [28].

## Tissue Distribution of Human Defensins

Six human α-defensins have been extensively characterised and are further subcategorised according to their expression patterns and gene structures, with α-defensins 1–4 being termed Human Neutrophil Peptides (HNPs) and α-defensins 5 and 6 being termed human (enteric) defensins (HDs) [8, 28–30].

HNPs are located within the azurophilic granules of human neutrophils and released through restricted secretion, whereby they fuse with phagolysosomes and directly kill phagocytosed microbes [8, 28–30]. Following holocrine secretion and infiltration of neutrophils during the inflammatory response, HNPs are also released into the extracellular milieu [30, 31]. HD5 and HD6 differ from the above-mentioned HNPs due to their expression and subsequent secretion by Paneth cells at the bottom of the small intestinal crypt [30].

In 1980, the first human α defensins HNP1–4 were isolated from myeloid cells. These were found to comprise 5–7% of the total neutrophil protein but were most prevalently located within the azurophilic granules at a much higher concentration of 30–50% [32]. HD5 and HD6 were discovered in 1992 and 1993, respectively, and identified in intestinal Paneth cells [31]. HD5 was later also found in the female reproductive tract [31]. Concentrations of HDs at the luminal surface of the small intestine are estimated to range between 50 and 250 µg/mL but significantly lower at the colonic mucosal surface due to the physical distance from the site of secretion [33].

The first human β defensin (HBD) was purified from plasma in 1995 [34], where its expression was traced back to epithelial cells of the kidneys, female reproductive tract [35], pancreas, and tissues such as those of the gingiva, parotid gland, buccal mucosa, and the tongue [27]. In 1997, HBD2 was discovered following extraction from psoriasis scales, it was found to be similarly expressed in the foreskin, trachea, and gingival mucosa [37]. Expression of HBD2 was also present, although to a much lesser extent, in the kidneys, uterus, and salivary glands [38]. HBD3 was discovered in 2000, following psoriasis scale extraction [36]. Using the computational Basic Local Alignment Search Tool (BLAST), the HBD3 gene was discovered [39], which soon allowed for the identification of expression of HBD3 in the skin, tonsils, epithelium of respiratory and reproductive tracts [36], adult heart tissue, skeletal muscle, placenta, foetal thymus, oesophagus, and trachea [39]. Following this discovery, the use of BLAST also allowed for the discovery of the HBD4 gene, the expression of which was found to be high in the testes and gastric antrum. Small amounts were also expressed in the uterus, neutrophils, thyroid gland, lung, and kidney tissue. In 2002, HBD5 and HBD6 were discovered and cloned, identifying the specific expression of these β-defensins in the human epididymis. During this year, new β-defensin genes were also discovered using computational search tools based on the hidden Markov models (HMMER) in combination with BLAST. Published findings in 2002 detail the analysis of five novel HBD transcripts (HBD25-29) existing mainly in the male genital tract and a few other organs [40]. Interestingly, none of these novel β-defensins were expressed in the trachea or the skin [41].

## Structure of Human Defensins

Human defensins (HDs) are further subcategorised according to the disulphide bond topology expressed. In α-defensins, the disulphide bonds are positioned between CysI–CysVI, CysII–CysIV, and CysIII–CysV. Typically, this group of defensins ranges between 29 and 50 amino acids in length. HNP1 and HNP3 are both comprised of 30 amino acids, with the only differing residue being at the N-terminus. HNP2 is distinctly post-translationally modified to remove the amino-terminal residue of HNP1 or HNP3. HNP2 is also the only α-defensin without a defined gene, although this could be due to its expression as HNP1 or HNP3 and then subsequently being post-translationally modified. HNP4 is slightly longer, composed of 35 amino acids and differs greatly from HNP1–3, only sharing 32% homology with the previously mentioned defensins. HD5 and HD6 are longer in chain size, with 45 and 50 amino acids, respectively and share less homology with previously mentioned defensins at 16.7%.

Alternatively, β-defensins are all 41–50 amino acids long with defined disulphide bonds positioned between CysI–CysV, CysII–CysIV, and CysIII–CysVI. The spacing between cysteine residues differs when comparing HBD1–3 against HBD4–6 and HBD25–29, whereby HBD1–3 has one greater amino acid between the second and third cysteine of the peptide, potentially altering the topology of the tertiary structure of these defensins. Additionally, this difference also suggests that undiscovered β-defensins may have similar spacing variations. Due to the differences in spacing, this also infers a difference in the tertiary structures of β-defensins, further implying a difference in the modes of action of these defensins against their targets.

As demonstrated above, there is quite a distinct structural variation between both α- and β-defensins, not only as groups but also as individual defensins. However, what remains constant in the structure of defensins is the consistently present three β-sheets and disulphide bond stabilised structure of these small amphipathic molecules [27].

### Multiple Pairwise Sequence Alignment and Structural Alignment of Human Defensins

Multiple pairwise sequence alignment studies and structural alignment studies were conducted on both α-defensins and β-defensins to identify and compare the similarities, differences, and evolutionary relationships among the sequences and structures of the two groups of human defensins.

#### α-Defensins

**Fig. 1:**
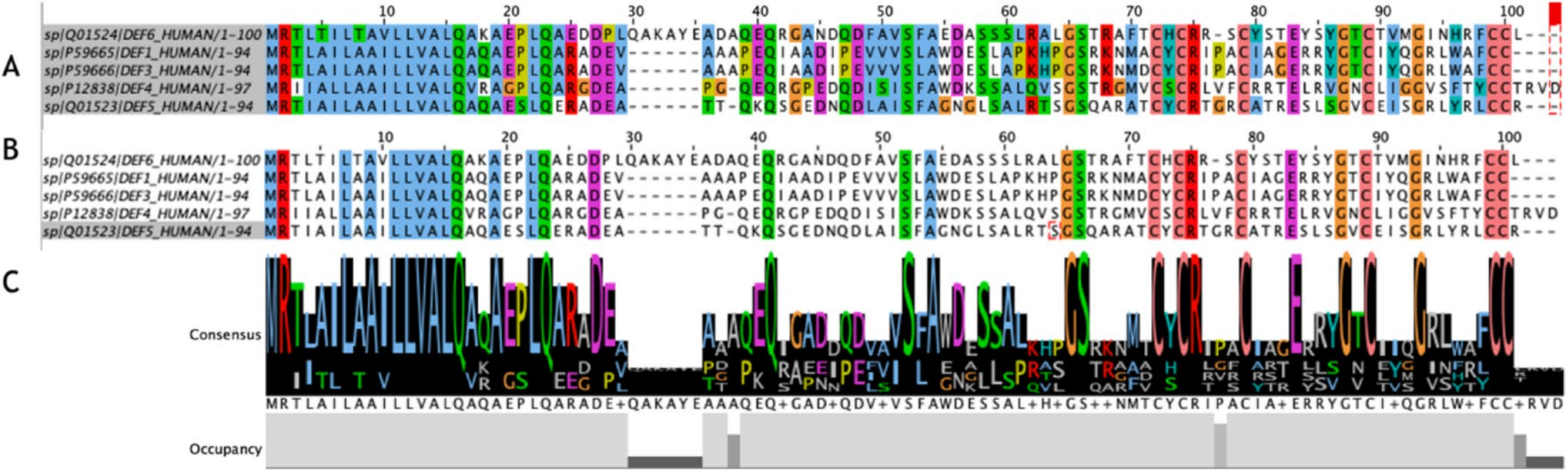
**A** Multiple sequence alignment study results of the human α-defensins HNP1, HNP3, HNP4, HD5, and HD6 amino acid sequences colour-coded in correspondence with the ClustalX defined colour scheme. HNP2 is not present in the sequences as it is post-translationally modified from HNP1 or HNP3 with no defined gene of its own. **B** The pairwise sequence alignment study results of the human α-defensin amino acid sequences filtered according to a > 90% conservation increment, and colour-coded in correspondence with the ClustalX defined colour scheme. **C** The consensus sequence of the pairwise sequence alignment study of the human α-defensins amino acid sequences indicated by the relative number of residues per column as visibly demonstrated by the size of the logo. Sequences were obtained from the UniProt database and included signal, peptide, and propeptide sequences

Multiple pairwise sequence alignment studies and structural alignment studies were conducted on both. Figure 1A demonstrates the results of multiple pairwise sequence analysis studies on the human α-defensins HNP1–4, HD5, and HD6 amino acid sequences. Many of the conserved sequences are clustered at the beginning of the sequence at positions 1–30, whereby these sequences were composed of largely hydrophobic (blue), polar (red, magenta, and green), aliphatic, and aromatic (cyan) residues. Additionally, the six cysteine residues that are a hallmark trait of the α-defensins and comprise the cysteine bridges that stabilise these proteins are conserved in all six defensins and found towards the end of the sequence with a 100% conservation increment. Figure 1B demonstrated the same sequences where the highlighted residues included are those with a conservation increment above 90%. Supplementary Fig. 2 contains the same sequences highlighted at all conservation increments from 20 to 100%. Of the 29 residues meeting this conservation threshold, 38% are hydrophobic residues (M, L, A, V), 6.9% are positively charged residues (R), 17.2% are polar residues (Q, S), 6.9% are negatively charged residues (D, E), 10.3% are glycines (G), and 20.6% are cysteines (C). Figure 1C provides a graphical representation of the residues conserved, as indicated by the size and height of the amino acid logo in the image, to further highlight the conserved residues within the sequences.

Furthermore, the cationicity of human α-defensins has been strongly linked to their functionality, as demonstrated by the supplementary Table 1 highlighting the percentage of cationic residues found in each α-defensin, as well as supplementary Fig. 1 which demonstrated the highly cationic electrostatic map of each human α-defensin. Additionally, the results of the multiple sequence alignment study highlighting the hydrophobic residues, which have been found to have a key role in the function of defensins can be found in supplementary Fig. 3.

**Fig. 2:**
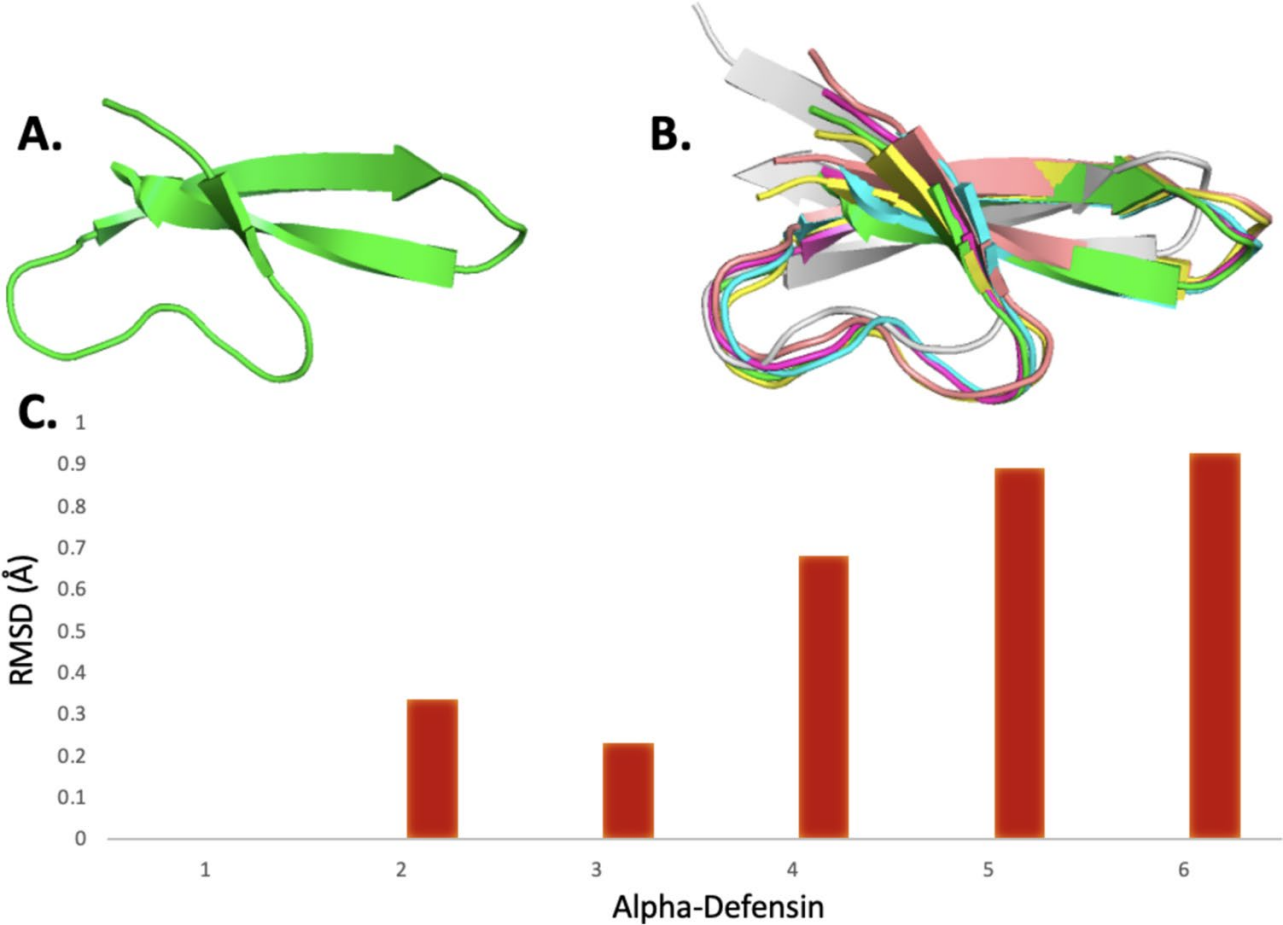
The results of a structural alignment study carried out on α-defensins HNP1(green), HNP2 (cyan), HNP3 (Pink), HNP4 (yellow), HD5 (blue), and HD6 (grey), whereby a clearly conserved region between all six defensins is visible in the 3D structural rendering. This image is generated using PyMol. **A** The structure of HNP1, **B** the structural alignment study of HNP1–4 and HD5–6, and **C** RMSD values of α-defensins when compared against HNP1

Findings of the structural alignment study seen in Fig. 2 carried out on the same human α-defensins correlate with the findings of the multiple sequence alignment study, whereby a clear conserved region is visible in the 3D representation of the defensins. The root mean square deviation (RMSD) values were calculated to measure the alignment of different α-defensins against HNP1, as seen in Fig. 2C. These values demonstrate good conservation within the structures as they are all aligned with an RMSD value of 1 Å or less, with HNP3 sharing the most similarity of the α-defensins.

#### β-Defensins

**Fig. 3:**
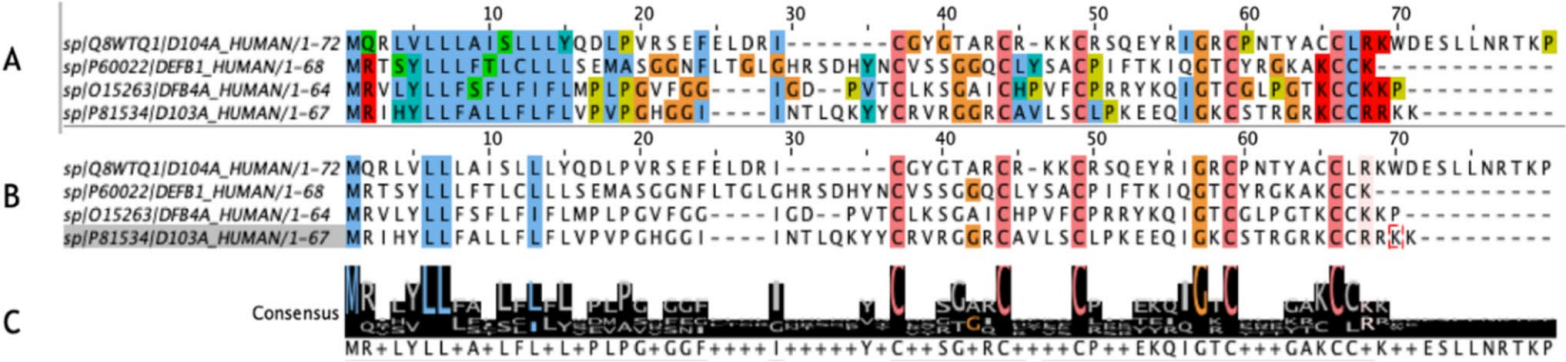
**A** Multiple sequence alignment study results of the human β-defensins HBD1–4 amino acid sequences colour-coded in correspondence with the ClustalX defined colour scheme. **B** Multiple sequence alignment study results of the human β-defensins amino acid sequences filtered according to a > 90% conservation increment, and colour-coded in correspondence with the CLUSTAL X defined colour scheme. **C** The consensus sequence of the pairwise sequence alignment study of the human β-defensins amino acid sequences as indicated by the relative number of residues per column as visibly demonstrated by the size of the logo

Unlike α-defensins, there are no other clusters of conserved sequences throughout all four β-defensins, showing an increased level of variation that is not present in the α-defensin counterparts. As seen in Fig. 3A, the conserved sequences present could be placed into two groups: hydrophobic amino acids (blue) conserved at the beginning of the sequence and the glycine residues (orange) and conserved cysteine residues (pink) that define defensins and compose the cysteine bridges that are hallmarks of their structures and located at the end of the sequence. The strongly conserved presence of hydrophobic residues in the same positions in both α- and β-HDs indicates the importance of these buried residues in forming the core of structural stability of these small proteins. Additionally, the conserved cysteines that define this group of defence peptides and give these proteins their defined structures also demonstrate the importance of these amino acids at these positions in shaping the structural integrity of the HDs. The role of the conserved glycines in the structures of both α- and β-defensins at the same positions is also strongly indicated to be present at the extremities of well-structured β strands and α helices, further supporting the other conserved residues in shaping the structural integrity of these proteins. These results can be viewed in Fig. 3, where Fig. 3A demonstrates the conserved sequences, Fig. 3B highlights the conserved amino acids at a > 90% conservation increment, and Fig. 3C provides a graphical representation of the residues conserved.

**Fig. 4:**
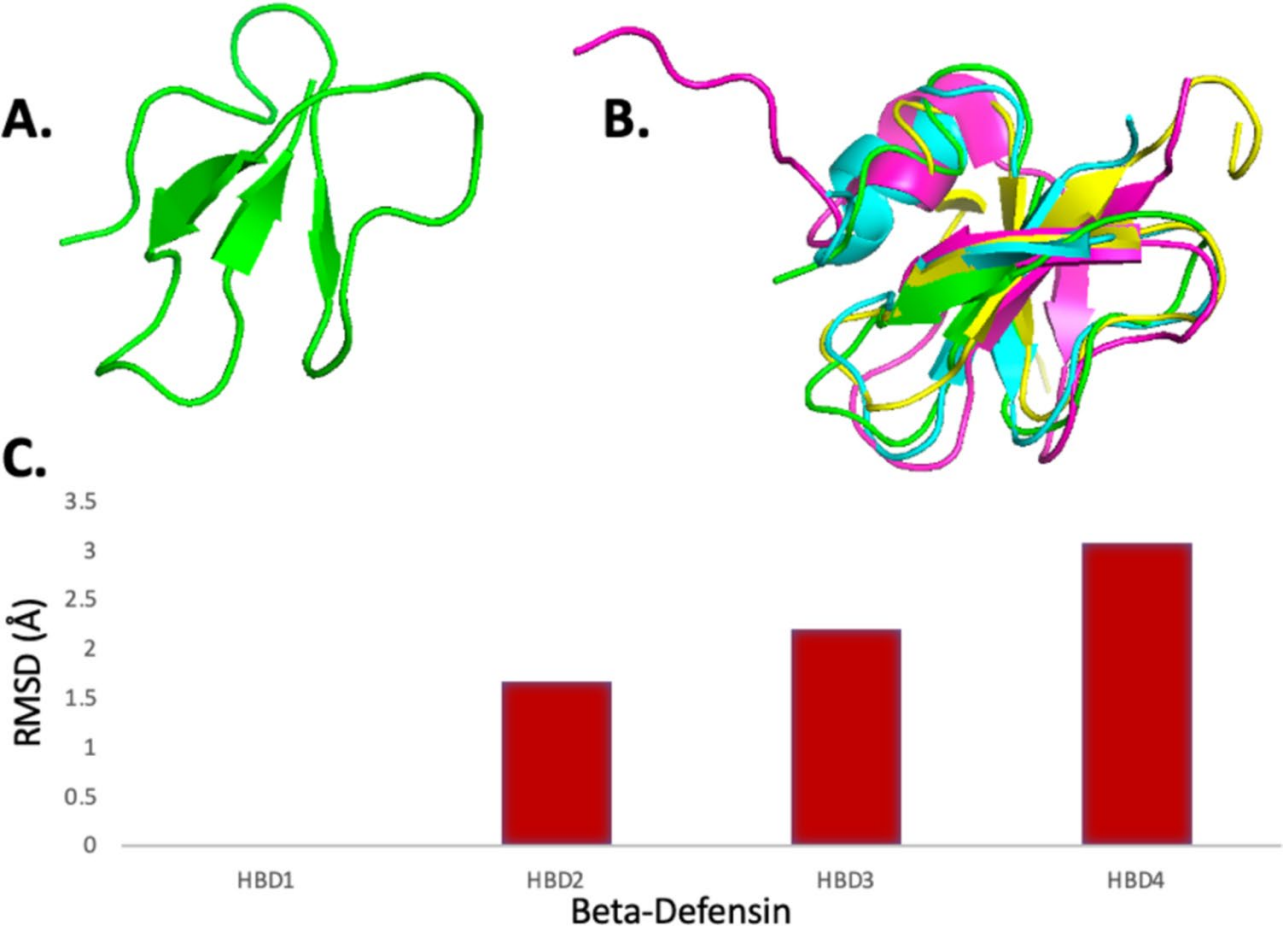
Demonstrates the results of the structural alignment study carried out on β-defensins HBD1(Green), HBD2 (Cyan), HBD3 (Pink), and HBD4 (yellow), whereby the conservation level is visible in the 3D structural rendering. This image is generated using PyMol. **A** The structure of HBD1, **B** the structural alignment study of HBD1–4, and **C** the RMSD values of β defensins when compared against HBD1

This lack of alignment is further demonstrated in the structural alignment study, as seen in Fig. 4, whereby a less explicit overlap in structural alignment is clear upon overlaying and aligning the four human β-defensins HBD1–4. The root mean square deviation (RMSD) values were calculated to measure the alignment of different β-defensins against HBD1, as seen in Fig. 4C. These values demonstrate less conservation within the structures as they are all aligned with RMSD values ranging between 1.5 and 3 Å, further corroborating that there is more variation between β-defensins compared to α-defensins.

## Gene Regulation of Human Defensins

Table 1 summarises the genetic regulation of all currently known human defensins (HDs) [41–43]. All HDs are found to be encoded by either chromosome 8 or 20, with the exception being HNP2, the only HD without an allocated gene location due to its generation being a direct result of the post-translational modification of HNP1 [41–43].

**Table 1:** Summary of the genetic regulation of human defensins, whereby details of gene location, size, exons, and introns are displayed where applicable

	Defensin	Gene location	Size	Exons	Introns
α-defensins	HNP1	DEFA1, 8p32.2-p23.1	3Kb	3 (5′ untranslated region, signal peptide/propeptide, mature peptide)	2
	HNP2	N/A	N/A	N/A	N/A
	HNP3	DEFA3, 8pter-p32.2	3Kb	3 (5′ untranslated region, signal peptide/propeptide, mature peptide)	2
	HNP4	DEFA4, 8P23	3Kb	3 (5′ untranslated region, signal peptide/propeptide, mature peptide)	2
	HD5	DEFA5, 8pter-p21	5.7kb	2 (signal peptide/propeptide, mature peptide)	1
	HD6	DEFA6, 8pter-p21	3kb	2 (signal peptide/propeptide, mature peptide)	1
β-defensins	HBD1	DEFB1, 8p23.2-p23.1	7kb	2 (signal peptide/propeptide, mature HBD1 peptide)	1
	HBD2	DEFB2, 8p23.1-p22	1.6kb	2 (signal peptide/portion of the propeptide, mature HBD2 peptide, and a short segment of propeptide)	1
	HBD3	DEFB3, 8p23	N/A	2 transcribed in the same direction as defb2	1
	HBD4	DEFB4, 8p23.1-p22	N/A	2 most of signal peptide/propeptide, the rest of signal peptide/propeptide	N/A
	HBD5	DEFB5, 8p22-8p21	N/A	3 transcribed in the opposite direction of HBD2-4	2
	HBD6	DEFB6, 8p23-p22	N/A		1
	HBD25	DEFB25, 20p13	N/A	2	1
	HBD26	DEFB26, 20p13	N/A	2	1
	HBD27	DEFB27, 20p13	N/A	2	1
	HBD28	DEFB28, 20p13	N/A	2	1
	HBD29	DEFB29, 20p13	N/A	2	1

### Gene Expression Regulation of Human Defensins

Expression of human defensins (HDs) can be both constitutive or inducible, with α-defensins being primarily constitutively expressed. HNP1–4 are typically synthesised before neutrophil maturation and differentiation, but following neutrophil cell maturation, they cannot be induced to produce these defensins [44]. HD5 and HD6 are similarly expressed constitutively by intestinal Paneth cells at a rate coinciding with their growth [31, 45].

On the other hand, β-defensin expression is induced and constitutively expressed [36, 39, 46, 47]. Inflammatory agents such as TNF-α, IL1-β, and LPS induce the expression of HBD2–4, although the exact mechanisms differ [36, 39, 48, 49]. For example, HBD2 expression is primarily mediated through the binding of NFκB to numerous binding sites at the intron and 5′ region of the HBD2 gene [48]. However, in the case of HBD3, IL1-β is responsible for the expression, demonstrating that HBD3 is induced through a different transcriptional regulatory pathway than HBD2 [36, 50]. In the case of HBD4, consistent stimulation with IL1-α, IL6, IFN-γ, or TNF-α did not appear to induce any levels of this defensin. However, when stimulated with PMA, levels of HBD4 increased over 60-fold [49]. Multiple NFκB binding sites are present in HBD6, and although the sequence of this code is defined within the gene (GGGRNTYC), it has not yet been functionally verified [43]. Neither HBD1 nor HBD5 have NFκB binding sites within the coding sequence. This highlights the varying pathways of HBD induction among this group of defensins.

## Antimicrobial potential of Human Defensins

**Fig. 5:**
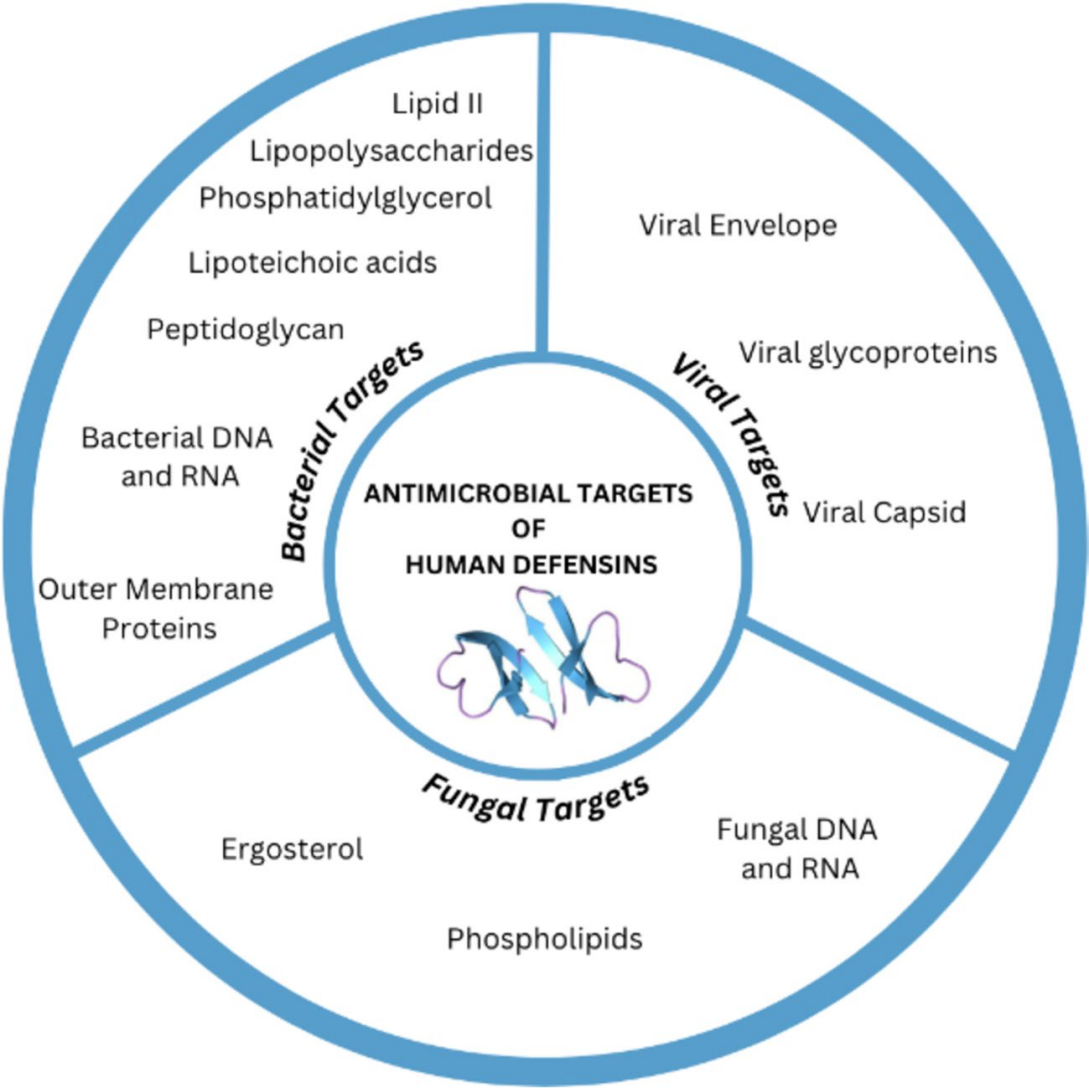
Visualisation of some of the molecular targets of human defensins across three categories: bacterial, viral, and fungal. Bacterial targets listed include lipid II, lipopolysaccharides, phosphatidylglycerol, lipoteichoic acids, peptidoglycan, bacterial DNA and RNA, and outer membrane proteins. Viral targets include viral envelope, viral glycoproteins, and viral capsid. Fungal targets include ergosterol, phospholipids, fungal DNA and RNA

Human defensins (HDs) have multifaceted roles in human defence against pathogens. The targets of their activities range across various species of bacteria, viruses, and fungi. Figure 5 visualises the molecular targets of these three categories.

### Antifungal Activity

Human β defensins 1–3 (HBD1–3) have been most extensively characterized, even so, little is known about the antifungal effects of human defensins. Z. Feng et al. [50] demonstrated that human β defensins act to limit fungal infection through hyphal induction, inhibition of adherence to epithelial cell membranes, and direct fungicidal activity [50].

HBD2 and HBD3 differ from HBD1 as they have no inhibitory effect on the Candida species C. glabrata. Treatment of *C*. *albicans* with HBD2 resulted in the thinning and rupturing of cell walls leading to possible cell lysis. Confocal microscopy attributed this effect to the *C*. *albicans* envelope being altered by the HBD2 [50]. It is worth noting that this study utilised HBD2 at concentrations 10 times greater than the IC_50_ which is difficult to be observed naturally under normal physiological conditions [50]. Similarly, the interaction of both HBD2 and HBD3 with *C*. *albicans* is energy-dependent, releasing ATP upon killing the fungus. Contrastingly, HBD3 activity was restored when sodium azide was added at high concentrations, indicating the potential for an alternative mode of action [50]. A study exploring the deletion of *C*. *albicans* cell surface heat shock proteins Ssa1p and Ssa2p revealed a reduction in the killing activity of both HBD2 and HBD3 when compared against wildtype *C*. *albicans*, indicating the requirement of these two proteins in the mode of action of these defensins against fungal infection [51].

### Antibacterial Activity

Human defensins (HDs) utilise two broad mechanisms to combat bacterial infection: through directly disrupting the membrane [8, 52, 53] or through the inhibition of bacterial cell wall synthesis [54]. HDs also play a role in reducing the burden of bacterial infection through the neutralisation of secreted bacterial toxins [55, 56] as in the case of human α-defensins demonstrating the ability to neutralize anthrax lethal toxin protecting against its fatal effects [57].

Due to the structural differences between human α and β defensins, there is a notable difference in the mechanisms utilised against bacterial infections. For example, as human α defensins are less cationic and more hydrophobic than their β defensin counterparts, the mechanisms by which they act against microbes also differ [8].

All bacterial membranes (Gram-positive and Gram-negative) comprise negatively charged phospholipids stabilised by bivalent cations such as magnesium and calcium [58]. Gram-negative bacteria differ from Gram-positive bacteria because they have an additional lipopolysaccharide-rich outer membrane [58]. Alternatively, human cells are richer in neutrally charged phospholipids. This difference in the charged membranes between negatively charged and positively charged bacterial and human cells allows for the high selectivity of antimicrobial peptides against bacteria [58, 59].

Classically, the functionality of defensins is through the disruption of the negatively charged, anionic bacterial membrane (Gram positive and Gram negative) [58, 60]. This disruption of the electrostatic forces keeping the membrane stable causes bacterial destruction [59, 60]. Three electrostatic models explain this effect on bacterial membranes: the barrel stave model, the carpet model, and the toroidal pore model [58, 59]. The barrel stave model refers to how peptides would be introduced perpendicularly in the bilayers before coalescing together to produce a pore [59]. Alternatively, the carpet model describes parallel absorption into the bilayers, which, after achieving sufficient coverage, generates a deterrent effect destroying the membrane [59]. Finally, the toroidal pore model suggests that peptides are introduced into the lipid bilayer perpendicularly, generating a regional membrane curvature that forms a pore by both peptides and phospholipid head groups [59].

Different mechanisms of antibacterial activity also exist, which are more specific to individual defensins. HNP1, for example, acts on the Gram-positive *S*. *aureus* via the restriction of lipid II, whereas it will act on the Gram-negative *E*. *coli* by destroying its cell membrane [61]. The broad activity of HD5 against both Gram-positive and Gram-negative bacteria has been attributed to its ability to increase bacterial membrane permeability. However, reports show it acts through its ability to bind to DNA, resulting in the inhibition of cellular replication [61]. Alternatively, in vivo studies of HD6 found protective effects against *Salmonella* infection despite little bactericidal activity being demonstrated in the in vitro studies of the same kind [62]. This mechanism is associated with the release of self-assembled nanonets, which interfere with the direct contact of the *Salmonella* bacterium with the intestinal epithelium by entrapping them within the nanonet structures [63].

HBD1 and HBD2 are both cationic, leading them to preferentially target Gram-negative bacteria [48]. However, of HBD1–3, HBD3 is significantly more cationic than HBD1 and HBD2, making it more potent as an antibacterial against both Gram-positive and Gram-negative bacteria in a structurally independent manner [36, 64–66]. Interestingly, following disulphide reduction, the HBD1 antimicrobial activity was found to go from weakly bactericidal to potently antimicrobial against both commensal Gram-positive bacteria and opportunistic fungal infection [67].

#### Human Defensins Against MRSA

Methicillin-resistant *Staphylococcus aureus* (MRSA) poses a formidable clinical threat due to its evolving resistant mechanisms against penicillin antibiotics [68]. MRSA infection in both clinical and community settings is a leading cause of septicaemia, endocarditis, skin, soft tissue, bone and joint infections and persistently contributes to a high rate of morbidity and mortality in both settings [68]. This rising issue needs to be urgently addressed through novel and innovative treatments capable of circumventing this evolving resistance [69].

The resistance in MRSA is attributed to multiple mechanisms; the presence of the mecA gene, which encodes an altered penicillin-binding protein (PBP2a) with a reduced affinity for β-lactam antibiotics [70, 71], efflux pumps that actively remove antibiotics from the bacterial cell [72], and the formation of biofilm to serve as a protective barrier against antibiotics.

The under-regulation of antimicrobial peptides, like human defensins (HDs), has been linked to the increased susceptibility of patients to opportunistic *S*. *aureus* cutaneous infections [73]. Conversely, the overexpression of these same peptides can further exacerbate inflammatory skin conditions such as psoriasis and rosacea [73].

The human β-defensins HBD1, HBD2, and HBD3 have been implicated in the innate immune response against *S*. *aureus*, including MRSA, validated by the expression of mRNA constitutively expressed in keratinocytes [66, 74]. Of the three HDs mentioned, HBD3 activity was the greatest against 44 clinical isolates of *S*. *aureus*, 22 of which were MRSA strains. However, of these MRSA strains, 55% exhibited more than 20% survival with HBD3, suggesting that there are still some resistance mechanisms against the activity of HDs. Inactivation of the *fmtC* gene, which is responsible for conferring methicillin resistance in MRSA, was found to increase the susceptibility of this bacteria to HBD3 action [74].

Another theorised method regarding the resistance of this bacteria to HDs is the production of a serine protease-like molecule staphylokinase (SAK) [75]. SAK was found to neutralise the bactericidal effects of human neutrophil peptides (HNPs). Cell-free SAK induces the secretion of HNPs, once released, SAK readily binds to the HNPs resulting in complex formation [75]. This mechanism effectively neutralises HDs, preventing them from adhering to the bacterial surface.

### Antiviral Activity

Human defensins (HDs) can reduce the infectivity of viruses from either enveloped or nonenveloped families [76]. Overall, three main methods exist by which HDs are able to combat viral infection through the direct interaction of the HDs with the lipid bilayer of a virion [77–80], the binding of specific HDs to glycoproteins and glycolipids [60, 79, 80], and direct protein–protein/protein–DNA interactions [81].

Inactivation of virions can be directly executed by binding HDs to the lipid bilayer of the viral envelope leading to effective neutralisation of the virus [82, 83]. This theory is supported by many studies which confirm that many enveloped viruses are effectively broadly neutralised due to the activity of HDs [81]. However, not all enveloped viruses are universally susceptible and the sensitivity of the viral infection to the activity of HDs is highly variable, with more negatively charged phospholipids being adversely affected when compared to neutral phospholipids, which go largely unscathed [84, 85].

The interaction between the viral envelope and HD triggers virions within the vicinity to aggregate, leading to another mechanism by which HDs act as antiviral agents. This aggregation greatly impedes cell binding and infiltration as it is more difficult for clumps of virions to invade through a cell wall [79, 86–88].

Another mechanism is the blockage of viral attachment receptor binding, which disrupts downstream processes required for cellular invasion [80, 89]. HDs also act by inhibiting the fusion or penetration of the viral genome into the host cell. For the viral genome to be inserted into the host cell, viruses utilise the six-helix bundle—a string of hydrophobic amino acids used to inject the lipid bilayer of the cell [90]. In the case of virions entering the cell, studies by Hazrati et al. [80] proposed that defensins can additionally block gene expression intracellularly, thus preventing the transcription, production, and assembly of viruses within the cell [80].

Diversity in the underlying inhibitory mechanisms exists amongst defensins, with different defensins employing different mechanisms, summarised in Table 2.

**Table 2:** Mechanisms of antiviral activities of different natural human defensins

	General mechanism of action	Type of defensin	Virus	Target/mechanism of action	Reference
1	Binding to viral particles	HNP4	HIV	Irreversible effect on virion infectivity by direct binding to viral particle	[91]
		HBD2, HBD3			[92]
		HNP1-3	HSV	Direct inactivation of the virus	[93]
		HNP1-2, HD5, HBD2	Influenza A	Aggregation of IAV and enhanced neutrophil-mediated clearance	[88, 94, 95]
		HNP1		Direct inactivation of the virus	[96]
		Mouse BD3	Influenza A	Direct interaction with viral envelopes, and the other involves indirect antiviral activity through interactions with potential target cells	[97]
2	Interfering with viral attachment to host cell receptors	HD5	HIV	Interfering with the reciprocal interaction between the envelope glycoprotein gp120 and CD4	[98]
3	Inhibition of viral entry	HNP4, HNP6, and HBD3	HSV	Prevent binding and entry	[80]
		HBD3	Influenza A	Blocking of viral fusion (fusion pore generation) by creating a protective barrier of immobilised surface glycoproteins from lectin-like properties of HBD3	[99]
		HBD2	RSV	Blocking of viral cellular entry, possibly because of the disintegration of the viral envelope	[100]
		HNP1	SARS-CoV-2	Destabilising SARS-CoV-2 spike protein and interfering with spike-mediated protein fusion in cell culture	[101]
				Inhibiting viral fusion but not the binding of the spike receptor-binding domain to hACE2	[102]
		HBD-2	SARS-CoV-2	Binds to the CoV-2-receptor binding domain (RBD) (KD ~ 300 nM), preventing it from binding to ACE2-expressing cells	[103]
		HD5(1–9)	HCMV	Inhibits HCMV cellular attachment and thereby entry	[104]
4	Blocking of uncoating by binding to the capsid	HD5	HPV	Prevention of the dissociation of the viral capsid from the genome	[105, 106]
			HSV	Enhanced binding to the capsid protein	[107]
		HD5	hAdV	Blocking of uncoating by binding to the capsid	[105]
5	Other mechanisms	HNP1	Influenza A	Inhibition of IAV replication through the inhibition of protein kinase C (PKC) activation in infected cells	[108]
		HD5	HPV	Blocking of a critical host-protease-mediated processing site of the minor capsid protein	[109]
		HNP1-3	HSV	Effective during the post-penetration period	[110]
		HBD2, HBD3	HIV	Inhibition of R5 and X4 HIV infection at a physiological concentration in the oral cavity by a mechanism not involving fusion inhibition or co-receptor modulation	[111]

Over the past two decades, many defensins have been investigated for their antiviral activities both in vitro and in vivo. For example, both Doss et al. [112] and Kota et al. [100] demonstrated that HBD2 neutralised the infectivity of IAV, RSV, and human parainfluenza virus (HPIV-3). Additionally, HBD2 diminished VZV replication in HaCat cells, a keratinocyte cell line, as demonstrated by Hazrati et al. [80] and Scudiero et al. [113]. Furthermore, HBD1–3 were shown to render HIV-1 virion particles less infectious. Interestingly, this effect was amplified when adding HBD2 and HBD3, as opposed to adding these peptides separately [114]. This effect was later confirmed by Bharucha et al. [115] where combined HBD2 and HBD3 were found to inhibit HIV replication dose-dependent on human monocyte-derived macrophages.

Regarding SARS-CoV-2, preincubation of pseudovirus particles of SARS-CoV-2 with HNP1 one hour before infection of human lung H1299 cells significantly inhibited the viral infection. In addition, both HNP1 and HD5 block SARS-CoV-2 infection at the viral entry step on HEK293T-hACE2 cells [102].

HD5 and its derivatives have also been evaluated in a lethal murine model of HSV-2 whereby HD5 demonstrated strong anti-HSV-2 activity for both prophylactic or therapeutic use, increasing the survival rates (60% and 70%, respectively) compared to non-treated controls (10%) [116]. HD5(1–9) also showed antiviral activity at concentrations of 50 µM in 318 HFF cells and 25 µM in ARPE-19 with an IC_50_ of ~ 40 µM in ARPE-19 [104]. Bastian and Schafer [117] also demonstrated the reduction of adenoviral infection by more than 95% if HNP1 is administered at 50 µg/mL with an IC_50_ of 15 µg/mL. In the case of IVA-infected mice with MBD-3, the protection rate was about 70% [118].

#### Human Defensins Against COVID-19

The COVID-19 pandemic emerged in late 2019 from the Wuhan region of China, resulting in an estimated 777 million infections and ~ 7 million deaths worldwide [119].

Currently, two major strategies are used against the SARS-CoV-2 virus: vaccinations and antivirals. Among the vaccines currently approved for use against COVID-19, three classifications of vaccines are in use: messenger ribonucleic acid (mRNA) vaccines (Pfizer-BioNTech, Moderna), adenovirus vector-based vaccines (AstraZeneca, Oxford University), and inactivated virus-based vaccines (Novavax) [120]. While these vaccines offer great preventative qualities in reducing the number of infections, the rapid rate of mutation with the generation of new COVID-19 variants such as α (alpha), β (beta), **γ** (gamma), δ (delta), omicron, and others, necessitates the urgent need for effective treatments once patients are infected and unprotected [121]. Additionally, the rapid development of new variants necessitates the need for constant booster immunisations, which is difficult to implement at a public health scale.

Different agents have been assessed for use against SARS-CoV-2, including general antiviral agents (nirmatrelvir plus ritonavir, sotrovimab, molnupiravir) [122], repurposed drugs (remdesivir, molnupiravir, baricitinib, anakinra, and tocilizumab), monoclonal antibodies targeting viral spike proteins [123] as well as antiviral peptides. The FDA also approved the emergency use of the two antivirals molnupiravir and paxlovid, by Merck and Pfizer, respectively, as further options for use against patients infected and at high risk of developing severe COVID-19. These agents significantly reduced the risk of severe disease progression and similarly improved the clinical outcomes of hospitalized patients [122]. Nevertheless, with the high mutation and fast replication rates of the virus, it remains imperative that new agents with novel mechanisms of action are found to control this pandemic.

Alongside the major antiviral drugs currently used to treat COVID-19, several antiviral peptides have demonstrated various potencies against coronaviruses [124]. These peptides have a broad range of targets capable of circumventing resistance mechanisms to current antiviral drugs, which is essential as the COVID-19 virus mutates rapidly. Some of these antiviral peptides target and induce the destruction of the viral envelope (murcoporin-M1) [125], target the viral spike (S) protein to inhibit fusion or target the S2 subunit [126]. An alternative method is targeting host proteins to interfere with the ability of the COVID-19 virus to bind or multiply within the host [127]. These mechanisms include the binding of the host angiotensin-converting enzyme-2 (hACE2) receptor, inhibiting late endosomal acidification, preventing the release of viral RNA (p9), and activation of protective host immune responses (RTD-1) [128]. This demonstrates the wide array and range of novel mechanisms targeting the SARS-CoV-2 virus on multiple fronts, circumventing the rapid mutational rate of the virus while offering protection and treatment. While these antiviral peptides show a lot of promise, there remains a significant delivery issue as peptides are sensitive to protease and peptidase activity, resulting in poor oral absorption. Additionally, the broad action of these peptides poses a risk of off-target toxicity. Once these issues are addressed and refined through the drug design process, they remain to be a potentially great resource to utilise against SARS-CoV-2.

The abundance of HDs in the systemic circulation (human neutrophil peptides HNP1 to 3), intestinal cells (HNP4 and 5), epithelial cells, and keratinocytes offers a wide range of local and systemic protection [1]. The antiviral activity of defensins was first reported in 1986 [129] and since then, their activities have been assessed against various enveloped and non-enveloped viruses, as shown in Tables 2 and 3.

**Table 3:** List of the clinical trials addressing the efficacy and safety of human defensin use in humans

Type of defensin	Disease	Country	Objective	Status	Reference
N/A	Skin aging	Advanced Dermatology, LLC, Lincolnshire, Illinois, USA	Skin care creams are supplemented by defensins and supportive molecules for skin hydration and anti-aging properties	Completed	ClinicalTrials.gov Identifier: NCT02765763
DefenAge 8-in-1 BioSerum	Periorbital wrinkles	West Dermatology Research Center/Cosmetic Laser Dermatology, San Diego, California, USA	Evaluate the efficacy of DefenAge 8-in-1 BioSerum supplemented with the enhanced concentration of defensins (enhanced 8-in-1 BioSerum) in improving periorbital wrinkles	Recruiting	ClinicalTrials.gov Identifier: NCT05082766
N/A	Fungal infection	Assiut University, Egypt	Determine the antifungal effects of defensin, cathelicidin, and histatins and their effects on biofilm formation and resistant isolates	Not yet recruiting	ClinicalTrials.gov Identifier: NCT05368948
Ghrelin	Lung infection caused by cystic fibrosis	Royal Papworth Hospital (Cambridge, UK)	For the intravenous treatment of airway inflammation, chronic respiratory infection, and lung infection caused by cystic fibrosis	Phase II clinical trial (completed)	[133]
Human cathelicidin peptide (LL-37)	Chronic leg ulcers	ProMore Pharma	Administrated topically for the treatment of chronic leg ulcers	Phase IIb clinical (current)	[133]
Histatin	Oral candidiasis	N/A	Treatment of Oral candidiasis as mouthwash	N/A	[135]

The promising activity of defensins against viral infections [76] has encouraged researchers to consider their use against SARS-CoV-2. Defensins displayed both direct and indirect activity against different variants of SARS-CoV-2. The abundance of HD5 in intestinal cells was found to protect COVID-19 patients from severe GIT consequences of the viral infection [130]. The activity of the intestinal HD5 was attributed to its ability to bind and interact with the ACE-2 receptors found on the surface of the intestinal cells, thus preventing initial viral interactions and subsequent disease pathogenesis [127]. The molecular target of HD5 was further confirmed ex vivo in the same study by the failure of SARS-CoV-2 to bind with caco-2 cells pre-incubated with HD5. HD5 also inhibited viral entry into human renal proximal tubular epithelial cells [102], suggesting HD5 is a promising anti-SARS-CoV-2 candidate in the early stages of infection. Another study compared the activity of different α- and β-defensins against pseudo-type viruses expressing SARS-CoV-2 spike proteins. HNP1–3 and HD5 showed superior activity against SARS-CoV-2 compared to HNP4 and HD6 whereas β-defensins showed no activity. Interestingly, HNP1–3 and HD5 prevented viral infection when pre-incubated with intestinal and lung epithelial cells. Nevertheless, their activity was completely diminished when added post-infection, supporting the hypothesis of viral entry-blocking activity rather than direct antiviral activity [102, 130, 131].

Docking-based investigation of HNP2 and HD5 interactions with SARS-CoV-2 showed their tentative affinity toward ACE2, both defensins might block the viral infection via their interaction with specific viral binding legends responsible for viral interaction with ACE-2 receptors [132].

From these findings, we decided to shed some light on the HNP1–3 and HD5 modes of interactions with SARS CoV-2’s cellular entry mediators (i.e., RBD and ACE2) using in-depth in silico-based investigation, with the results found in section 8.

## Clinical Trials

Despite the great potential for the antibacterial, antifungal, and antiviral activities of natural defensins, their application as antimicrobials is still in its infancy. This is reflected by the limited number of clinical trials addressing their efficacy and safety on human beings (Table 3). Instead, a huge number of defensin-derived synthetic peptides, peptidomimetics, and defensin analogues have entered different phases of clinical trials [132–134].

## Disadvantages and Toxicity

Despite the great antimicrobial potential of natural human defensins (HDs), there are many hurdles towards introducing them in the pharmaceutical market. For instance, like all AMPs, HDs show humble in vivo stability owing to their sensitivity to the action of proteases, osmolarity, and differences in pH. In addition, the nonspecific action on the cell membrane of both bacterial and human cells evokes significant concerns regarding the safety of systemic HD use.

On an economic level, the high-cost production of HDs greatly limits the number of clinical trials testing their use under actual conditions. This could be observed by the small number of official active or completed clinical trials addressing the value of HD clinical use (Table 3).

Nevertheless, new strategies have been developed to tackle these challenges, including chemical modification of HDs to enhance their stability and safety through cyclization, halogenation, acetylation, or conjugation to nanocarriers.

Cyclisation can be used to address the issue of proteolytic degradation and instability found in many antimicrobial peptides. This process involves the transformation of a linear peptide into a cyclic peptide through one of three methods: the addition of a disulfide bond or the head-to-tail cyclisation through the addition of a beta-lactam moiety to connect the N- and C-termini of the peptide [136]. The increased resistance to proteolytic degradation through this method enhances the bioavailability and half-life of AMPs in vivo. Among its benefits is also the ability to improve the antimicrobial activity of the AMP due to an observable increase in membrane penetration and reduced susceptibility to conformational changes [136–138]. HDs are naturally cyclic antimicrobial peptides, and this is thought to contribute to the potent antimicrobial activity exhibited by this group.

Halogenation involves the addition of one or more of chlorine, bromine, fluorine, or iodine, and is another example of the methods used to enhance the properties of many AMPs. This method has been described as “transformative” with its ability to enhance stability, improve solubility, and increase antimicrobial activity and efficacy against target pathogens and has been applied to over a third of drugs undergoing clinical trials [139–141].

Nanomedicine is a relatively new field of science whereby nanoparticles are combined with drugs to improve different pharmacologic properties like delivery, stability, and target specificity [142–144]. The small size of nanoparticles provides a large surface area capable of loading drug molecules. Metallic nanoparticles made of silver and zinc oxide also have complimentary antimicrobial properties that can enhance the antimicrobial properties of AMPs [145–147]. Gold nanoparticles also provide a complimentary enhancement of the potency of AMPs while reducing toxicity and increasing serum stability [148]. Polymeric nanoparticles (poly lactic-co-glycolic acid) and magnetic nanoparticles are characterised by their low toxicity and sustained-release properties, making them useful in the design of an effective drug delivery system [149]. Furthermore, the conjugation of an AMP with a nanoparticle allows for the unique opportunity to design self-assembling compounds involving the spontaneous formation of stable structures, which can be controlled to give supramolecular compounds that dissemble upon contact with the bacterial target. These self-assembling AMPs have improved efficacy, biocompatibility, stability, resistance to proteolytic degradation, and lower toxicity. Lei et al. [150] were able to create self-assembling supramolecules of HD5 through the myristylation of the C-terminal, which resulted in significantly improved efficacy against MRSA and *E*. *coli* [150].

Additionally, the synthesis of peptidomimetics and the design of hybrid peptides provide another cheaper and more effective alternative for clinical and market use [59]. Peptidomimetics, synthetic molecules engineered to mimic structural and functional features of known peptides, are designed to display similar biological profiles as the parent peptide. They have enhanced structural and pharmacokinetic properties, making them more favourable alternatives to parent peptides alone [151]. This technique allows for modifying the physiochemical properties of peptides, such as cationicity, hydrophobicity, and amphipathicity, to transform parent peptides into more stable and potent antimicrobial agents. These peptidomimetics can be designed using numerous computational techniques where their potential and activity are screened and enhanced before they are synthesised and evaluated in vitro and in vivo [151].

While the techniques previously described in this section can all enhance the activity of AMPs and address the limitations that hinder these peptides from reaching the market, the refinement and enhancement of these peptides remain a considerable challenge, with more research being needed to fully unlock all their potential.

## In Silico Investigation of Human Defensins Against SARS CoV-2 Cellular Entry

As shown in the recently reported literature, HNP1–3 and HD5 can inhibit SARS-CoV-2 cellular entry by interacting with ACE2 (the main receptor of SARS-CoV-2). However, the exact modes of interaction at the molecular level were not elucidated. In this study, multiple in silico-based approaches were employed to elucidate and explain these interactions at a molecular level.

ACE2, as a membrane-bound receptor, presents a more accessible target for HDs. Its location on the cell surface makes it an ideal candidate for interaction with HDs, particularly in the context of blocking viral entry, a key mechanism of SARS-CoV-2 infection. Given that HDs are cationic antimicrobial peptides and typically function extracellularly, they are less likely to efficiently cross cellular membranes, which limits their interactions with intracellular targets.

### Docking Study

**Fig. 6:**
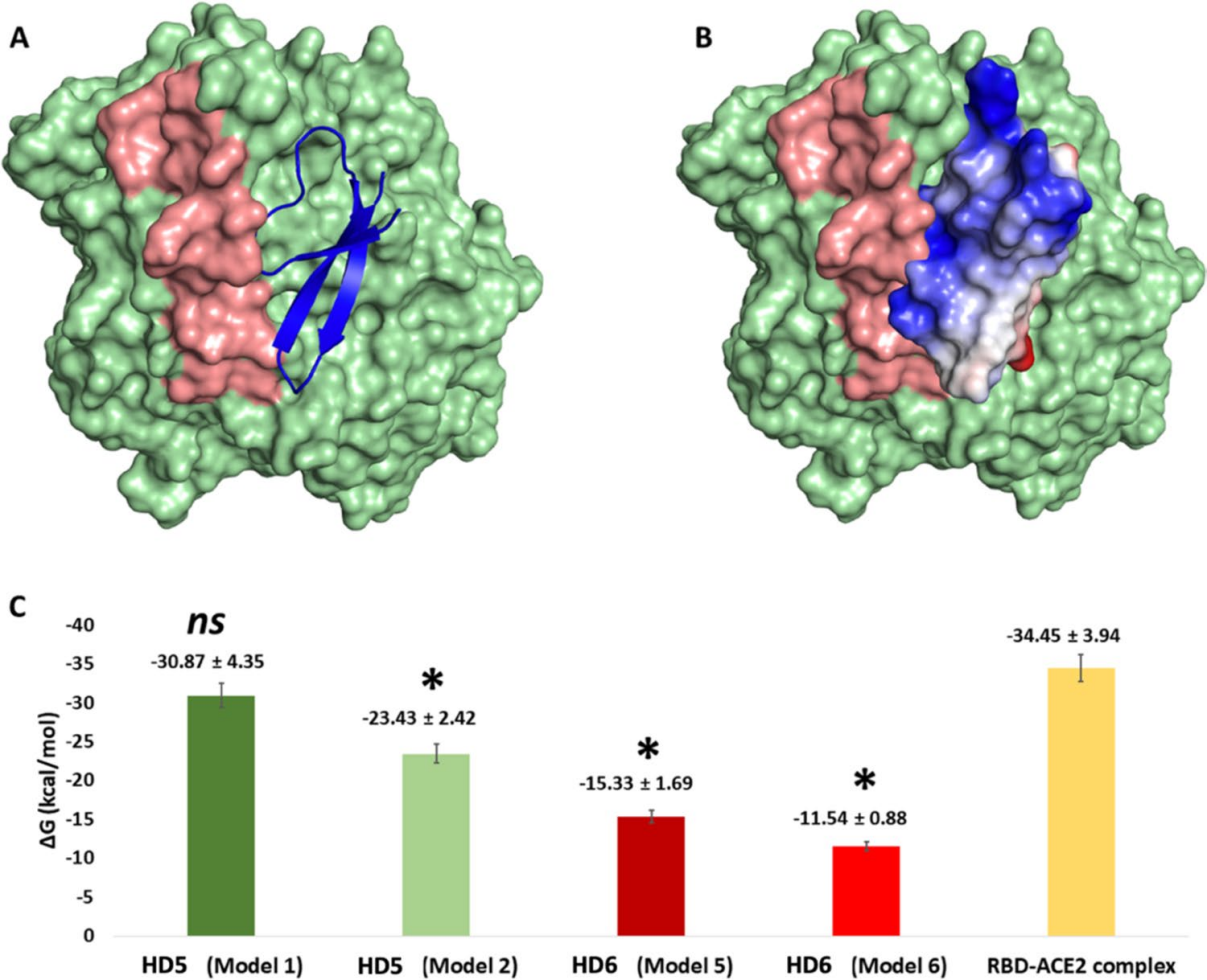
The binding mode of HD5 with ACE2 (Model 1) in blue cartoon representation (**A**) and van der Waals surface electrostatics representation (**B**) where blue regions indicate positively charged amino acid residues. The brick-red coloured region in **A** and **B** represents the RBD binding site on the green-coloured ACE2 structure. **C** Calculated Δ*G*_Binding_ between HD5 and HD6 and ACE2 in the selected modes (Models 1 and 2 for HD5 and Models 5 and 6 for HD6) and Δ*G*_Binding_ between RBD and ACE2. ns indicates no significant difference in comparison with RBD-ACE2; *p < 0.05, *n* = 3, significant difference in comparison with RBD and ACE2

The modelled structures of HNP1–4 along with HD5 and 6 were docked into the modelled structure of human ACE2 (hACE2) (see the supplementary file for more details about the docking method). For each docked defensin, ten models were generated and binding poses near the RBD binding site on ACE2 were selected for Molecular dynamic simulations (MDS)-based binding free energy calculation (ΔG Binding). The rationale behind these selections is that binding to the RBD binding site (Fig. 6) or near it will hinder the binding of SARS CoV-2 RBD, and hence, viral entry pathways will be compromised. Of the six human α-defensins, only HD5 and HD6 showed bindings models (2 models and 3 models, respectively) with the ACE-2 RBD binding site, leading to these models being selected for further investigation.

The selected models were subjected to 50-ns long MDS and binding free energy (ΔG Binding) calculations using the Molecular Mechanics Generalized Born Surface Area (MM/GBSA) method [152]. The main objective of this step is to identify the optimal binding model for each human defensin (HD) structure in terms of the binding affinity towards the RBD binding site of ACE2.

As shown in Fig. 6, HD5 (model 1) showed the best binding affinity toward ACE2 with a calculated Δ*G*_Binding_ value of − 30.87 ± 4.35 kcal/mol which was comparable to that of the RBD and ACE2 (Δ*G*_Binding_ = − 34.45 ± 3.94 kcal/mol).

HD5 in its complex with ACE2 in Model 1 (Fig. 6) showed great affinity towards the binding site that intersects with the RBD binding site, leading to the competitive inhibition of the naturally occurring binding with SARS CoV-2’s RBD, in turn, preventing viral entry into the host cells and slowing down the pathogenesis of the disease. Consequently, the HD5–ACE2 complex (Model 1) was subjected to longer MD simulation runs (300 ns long) to study the HD5 binding stability with ACE2 and to explore its dynamic binding mode (see the supplementary file for more details about the MD simulation).

As depicted in Fig. 6, HD5 showed stable binding with ACE2 throughout the simulation with an average RMSD of 1.7 Å. When compared against the RBD (average RMSD = 2.3 Å), HD5 showed fewer fluctuations, indicating slightly more stability than RBD.

**Fig. 7:**
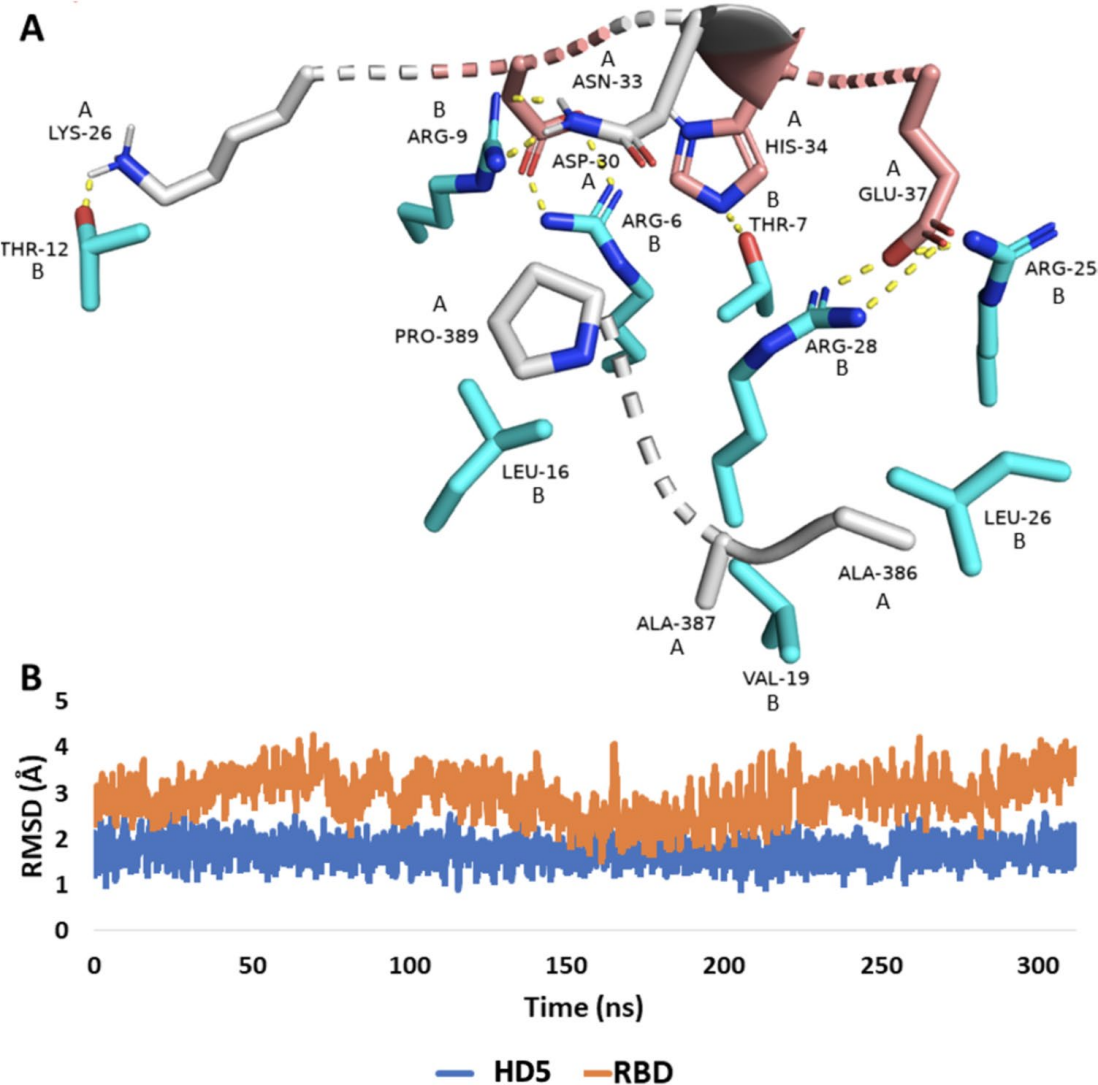
Dynamic binding mode of HD5 with ACE2 (Model 1) extracted from the MD simulation trajectory as the most populated pose. Amino acid residues assigned with the letter A are for ACE2 (white-coloured and brick-red coloured residues), while those with the letter B are for HD5 (cyan-coloured residues). Brick-red coloured residues belong to the ACE2’s RBD binding site [153]. **B** RMSD of HD5 in comparison with that of RBD over the course of 300-ns long MD simulation

Exploring the most populated pose for the MDS trajectory indicated that the stable binding of HD5 came from its multiple interactions with ACE2’s amino acid residues, notably the electrostatic interactions between the cationic residues of HD5 (e.g., ARG-6B; ARG-9B, ARG-25B, and ARG-28B) and the anionic residues of ACE2 (e.g., ASP-30A, ASN-33, GLU-37, respectively) (Fig. 7). Additionally, the H-bonds established between LYS-26A and THR12B, HIS-34A and THR-7B. Moreover, the hydrophobic interactions between ALA-386A and LEU-26B, PRO-389A and LEU-16B, ALA-387A, and VAL-19B.

It is worth noting that three of the ACE2’s interacting amino acid residues (e.g., ASN-33A, HIS-34A, and GLU-37A) belong to the RBD binding site, indicating that upon HD5 binding, these amino acid residues will be unavailable to bind with RBD. The previously described in silico-based findings point to the ability of HD5 to compete with RBD for ACE2, hence, hindering the SARS-CoV-2 attachment mechanisms preventing entry to the host cells, a conclusion further supported by the in vitro testing previously described [127].

## Study Limitations and Future Perspectives

Human defensins (HDs) play a multitude of immunomodulatory functions within the innate immune system, primarily acting to protect the host from infections. The past few decades have yielded many investigations into the potential antimicrobial activities of these defensins, both in vitro and in vivo, exploring the therapeutic use of this group of defence peptides. As HDs have been found to display both direct and indirect mechanisms against different viral targets, there has been considerable interest in exploring their potential use against SARS-CoV-2 infection.

In this study, our group has conducted docking-based investigations of both HNP2 and HD5 interactions with SARS-CoV-2. The results of this docking-based analysis showed the tentative affinity of these two defence peptides toward ACE2 receptors, pointing to the ability of HD5 to compete with RBD for ACE2.

The computational in silico studies conducted here provide a solid base and a good theoretical indication of the potential success of using a peptidomimetic compound based on the structural features of HDs to target the SARS-CoV-2 virus successfully. While in silico studies offer advantages such as cost-efficiency, speed, and the ability to explore theoretical scenarios, they have notable limitations compared to in vitro methods. Computational models rely on simplifications of complex dynamic systems, only capturing a simplified version of the complexity found in biological systems. Despite these limitations, the integration of in silico and in vitro approaches provides a more comprehensive framework for research and development. So, while these results are promising, they merely serve as a foundation for future in vitro and in vivo validation studies to further build on and validate the results discussed in this paper.

Despite the great antimicrobial potential of natural human defensins (HDs), there remain numerous hurdles towards introducing them in the pharmaceutical market, such as high-cost production and sensitivity/stability within the body as a drug formulation. For instance, the low stability of AMPs in vivo has been attributed to protease action and differences in osmolarity and pH. Additionally, the nonspecific mechanisms of action on cell membranes of bacterial and human cells pose a risk with systemic HD use. Nevertheless, these concerns can be addressed through the chemical modification of HDs to enhance their stability and safety and the synthesis of peptidomimetics and hybrid peptides.

Overall, of the human defensins, HD5, in particular, has untapped potential and shows great promise as a novel broad-spectrum antibacterial agent against both Gram-positive and Gram-negative bacteria, as well as potentially being developed as an antiviral agent against SARS-CoV-2. The in silico studies conducted by our group further support this theory, demonstrating the potential success of developing HD5 into an effective antimicrobial agent. These results, coupled with the current research found within the literature, can serve as a strong foundation for the successful design and synthesis of an antiviral peptidomimetic capable of successfully targeting SARS-CoV-2.

## Supplementary Information

Below is the link to the electronic supplementary material.

Supplementary file1 (PDF 1111 KB)

## Data Availability

All generated data is provided within the manuscript or supplementary information files.

## Abbreviations

ACE2: Angiotensin-converting enzyme 2
AMPs: Antimicrobial peptide
BLAST: Basic local alignment search tool
COVID-19: Coronavirus disease 2019
DNA: Deoxyribonucleic acid
GIT: Gastrointestinal tract
FDA: Food and drug administration
hACE2: Human angiotensin-converting enzyme 2
HBD: Human β-defensin
HBD1: Human β-defensin 1
HBD2: Human β-defensin 2
HBD3: Human β-defensin 3
HBD4: Human β-defensin 4
HBD5: Human β-defensin 5
HBD6: Human β-defensin 6
HDs: Human defensin
HD5: Human defensin 5
HD6: Human defensin 6
HNP1: Human neutrophil peptide 1
HNP2: Human neutrophil peptide 2
HNP3: Human neutrophil peptide 3
HNP4: Human neutrophil peptide 4
IC50: Half-maximal inhibitory concentration
IL1-β: Interleukin 1 β
kDa: Kilo Dalton
LDL: Low-density lipoprotein
LPS: Lipopolysaccharide
MDS: Molecular dynamics simulation
MRSA: Methicillin-resistant *Staphylococcus aureus*
NFκB: Nuclear factor κ B
PMA: Propidium monoazide
RMSD: Root mean square deviation
RNA: Ribonucleic acid
S: Spike protein
SARS-CoV-2: Severe acute respiratory syndrome coronavirus 2
TNF-α: Tumour necrosis factor α
