# Supplementary material for: Human Defensins: Structure, Function, and Potential as Therapeutic Antimicrobial Agents with Highlights Against SARS CoV-2
Source: Probiotics Antimicrob Proteins. 2024 Dec 18;17(3):1563–83. doi: 10.1007/s12602-024-10436-8 (PMC12055905; doi:10.1007/s12602-024-10436-8)
Supplement: Supplementary file 1 — Supplementary file1 (PDF 1111 KB) [file 12602_2024_10436_MOESM1_ESM.pdf]

Supplementary information

Human defensins: structure, function, and potential as therapeutic antimicrobial agents with highlights against SARS CoV-2

Table 1 This table demonstrates the percentage of cationic residues found in alpha defensins.

Human Defensin	Percentage of cationic residues
HNP1	8.51%
HNP2	8.51%
HNP3	8.51%
HNP4	7.22%
HNP5	6.38%
HNP6	7.00%

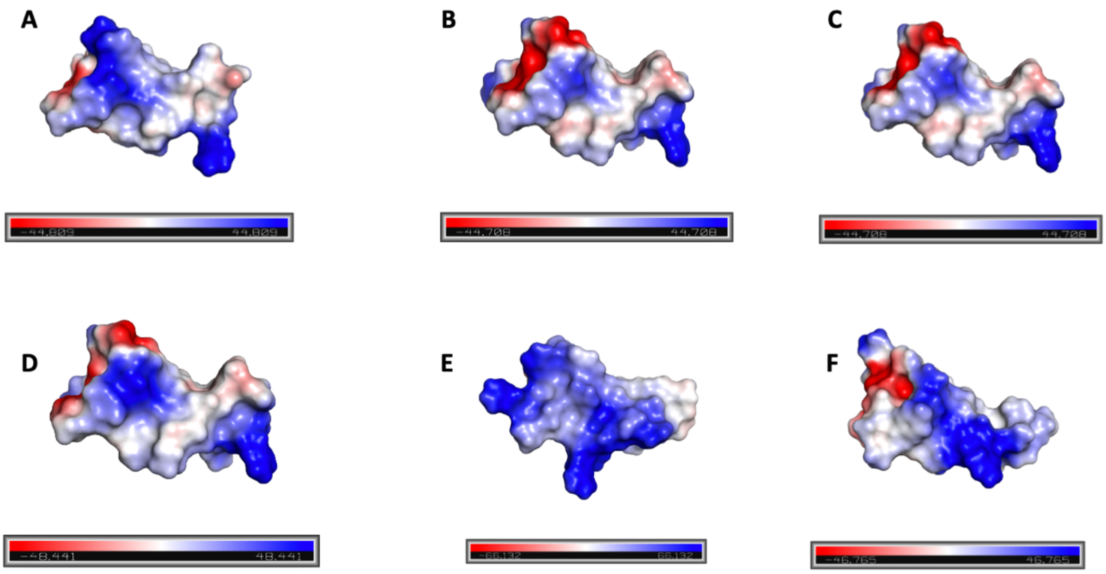

Supplementary Figure 1. This figure visually represents the electrostatic map of individual human alpha defensins (A =HNP1, B=HNP2, C=HNP3, D=HNP4, E= HD5, F=HD6) whereby the colour blue highlights cationic hotspots of the peptide and red represents anionic hotspots respectively.

2A.

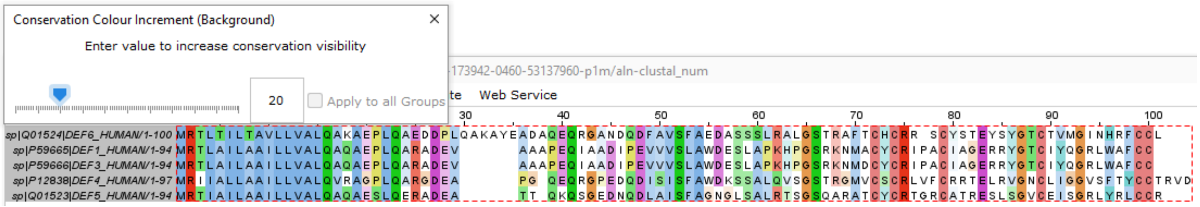

2B.

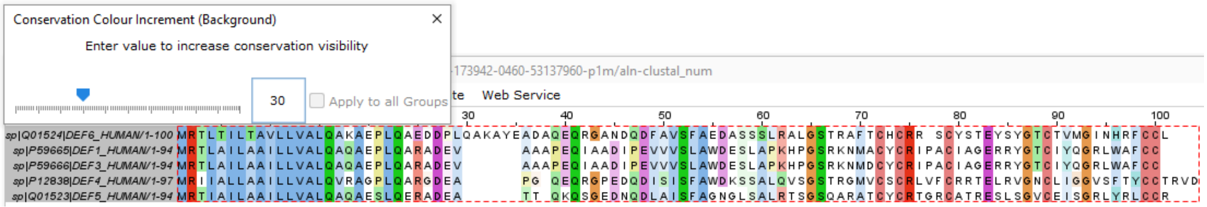

2C.

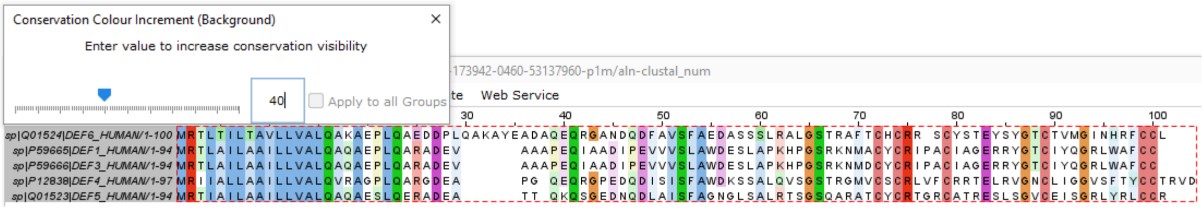

2D.

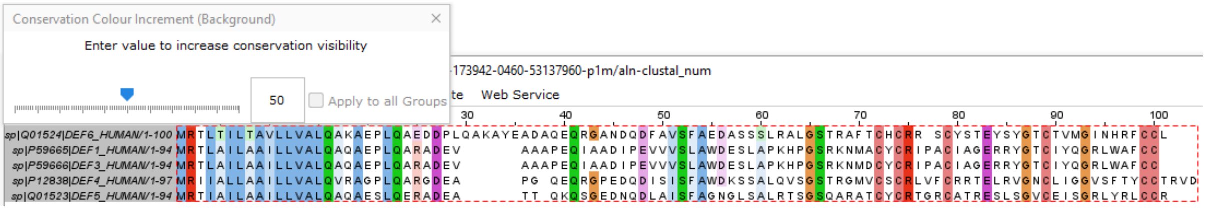

2E.

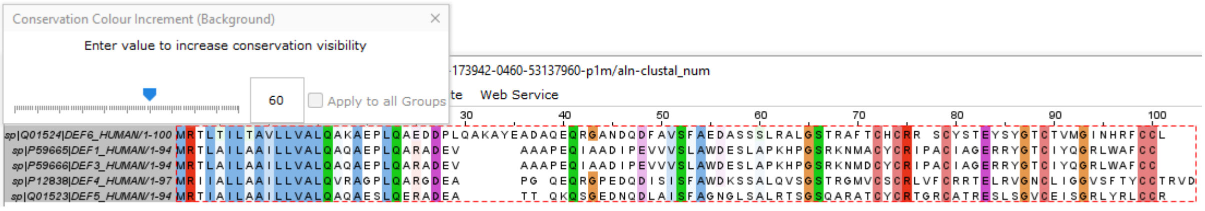

2F.

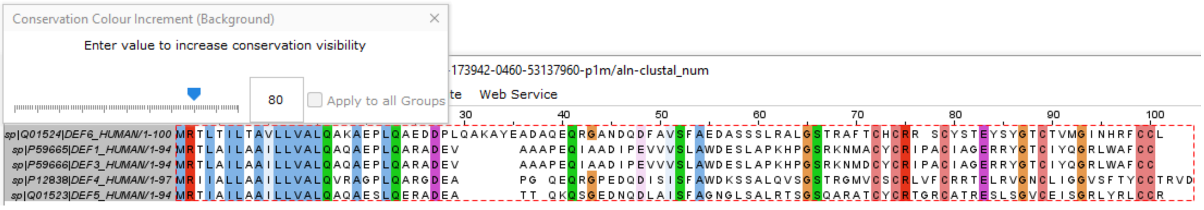

2G.

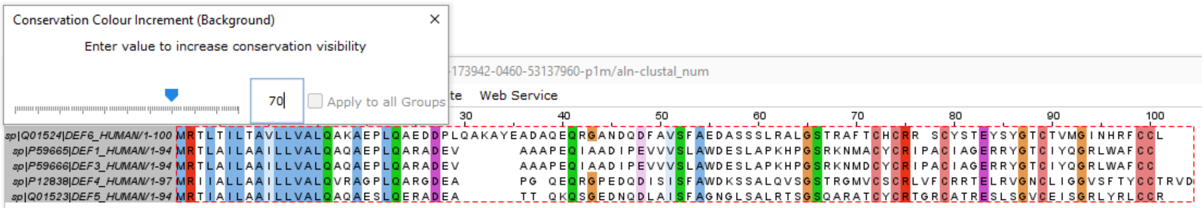

2H.

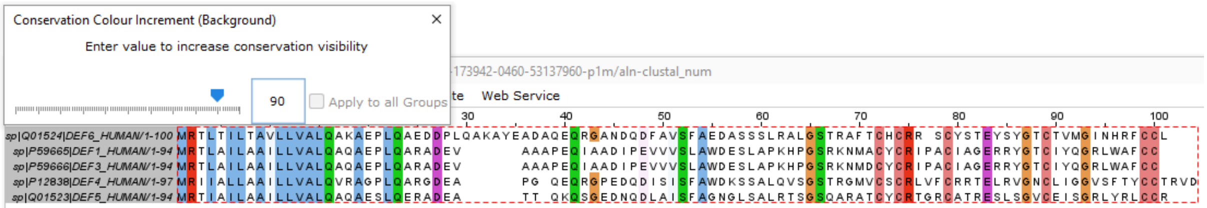

2I.

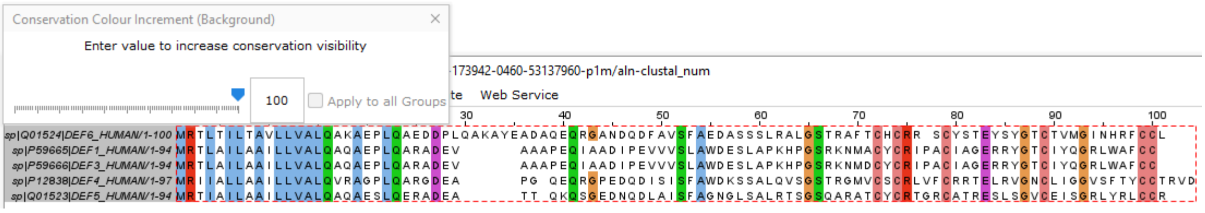

Supplementary Figure 2. This figure demonstrates the results of the multiple sequence alignment study conducted on the human alpha defensins whereby residues are highlighted and coloured according to the ClustalX colourscheme defined on the Jalview website. Figures 2A-2I demonstrate the results of filtering according to the conservation increment whereby the conservation increment is set at 20% for Supplementary Figure 2A, 30% for Supplementary Figure 2B, 40% Supplementary Figure 2C, 40% for Supplementary Figure2D, 50% for Supplementary Figure 2E, 60% for Supplementary Figure 2F, 70% for Supplementary Figure 2G, 80% for Supplementary Figure 2G, 90% for Supplementary Figure 2H and 100% for Supplementary Figure 2I, demonstrating an increasing level of conservation as the value of the conservation increment increases progressively.

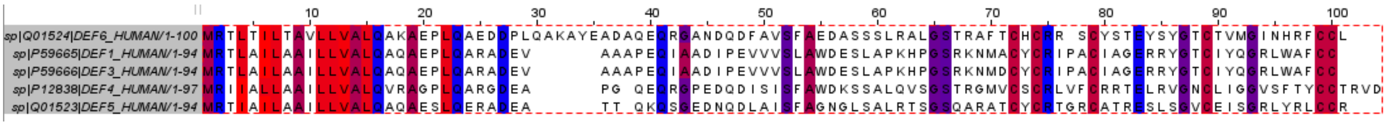

Supplementary Figure 3. This figure demonstrates the results of a mulitple sequence alignment study conducted on the human alpha defensins whereby hydrophobic residues are highlighted based on the colour scheme assigned by JalView which can be found in the Amino acid and Nucleotide based colour schemes listed on the website.

Methods

Molecular Docking

The protein docking study was carried out against both the receptor-binding domain (RBD) of SARS CoV-2′s spike protein (S-protein) and human angiotensin-converting enzyme-2 (ACE2) (PDB codes: 6lZG and 1R42, respectively) [1,2]. An Autodock Vina docking machine [3] was used for docking experiments. To determine the best binding site for docking, we docked the prepared structures of human defensins (HD1-HD6; PDB ID: 3gny, 1zmk, 1dfn, 1zmm, 1zmp, and 1zmq, respectively) against both S-protein and ACE2 using the ClusPro server [4] by uploading each crystal structure to the website and choosing heparin as a ligand. The retrieved docking poses were then arranged from the highest to the lowest scores. We selected the docking pose of the highest score and subjected it to a short molecular dynamic simulation (25 ns) experiment to study the stability of each complex in terms of the RMSD profile over the course of simulations. All results were visualized using PyMol software [5].

Molecular Dynamic Simulation

MD simulation experiments were carried out using the MDS machine in Maestro software, Desmond v. 2. [6-8], using its default force field (i.e., OPLS-AA). Protein–ligand systems were constructed using the System Builder function. Thereafter, these systems were embedded in an orthorhombic box consisting of TIP3P water and 0.15 M Na^+ and Cl^- ions (the default dimensions were used). Subsequently, the prepared systems were energy-minimized and equilibrated for 10 ns. Ligand parameterization was carried out during the system building step according to the OPLS force field. For MD simulations carried out using NAMD software, the parameters and topologies of the ligands were computed using the Charmm36 force field. The online software Ligand Reader and Modeler (http://www.charmm-gui.org/?doc=input/ligandrm) [9] and the VMD plugin Force Field Toolkit (ffTK) [10] were utilized for this regard. The generated parameters and topology files were then loaded into VMD to read the protein–ligand complexes without errors, then the simulation step was performed. MD simulations were run for 50 ns at 310 K in the NPT ensemble with the Nose–Hoover thermostat and Martyna-Tobias-Klein barostat using anisotropic coupling. We selected the best binding poses for each compound as starting co-ordinates to investigate their binding stability and mode of interaction.

Binding Free Energy Calculations

Binding free energy calculations (ΔG) were performed using the free energy perturbation (FEP) method [11]. Briefly, this method estimates the binding free energy (i.e., ΔGbinding) according to the following equation: $\Delta G_{\mathrm{binding}} = \Delta G_{\mathrm{Complex}} - \Delta G_{\mathrm{Ligand}}$. These estimations were derived from separate simulations (NAMD software was used for these experiments). All input files required for simulation by NAMD were papered using the online website Charmm-GUI (https://charmm-gui.org/?doc=input/afes.abinding). Subsequently, we loaded these files into

NAMD in order to produce the required simulations. The FEP function in NAMD was used to accomplish this experiment. The equilibration step was achieved in the NPT ensemble at 300 K and 1 atm (1.01325 bar) with Langevin piston pressure (for ”complex” and ”ligand”) in the presence of the TIP3P water model. Then, 10 ns FEP simulations were carried out for each ligand and the last 5 ns of the free energy values was measured for the final free energy estimation [11]. All resulting trajectories were visualized and analyzed by VMD software.

References

1. Wang, Q.; Zhang, Y.; Wu, L.; Niu, S.; Song, C.; Zhang, Z.; Lu, G.; Qiao, C.; Hu, Y.; Yuen, K.-Y.; et al. Structural and Functional Basis of SARS-CoV-2 Entry by Using Human ACE2. Cell 2020, 181, 894–904.

2. Towler, P.; Staker, B.; Prasad, S.G.; Menon, S.; Tang, J.; Parsons, T.; Ryan, D.; Fisher, M.; Williams, D.; Dales, N.A.; et al. ACE2 X-ray structures reveal a large hinge-bending motion important for inhibitor binding and catalysis. J. Biol. Chem. 2004, 279, 17996–18007.

3. Mottarella, S.E.; Beglov, D.; Beglova, N.; Nugent, M.A.; Kozakov, D.; Vajda, S. Docking server for the identification of heparin binding sites on proteins. J. Chem. Inf. Model 2014, 54, 2068–2078.

4. Kozakov, D., Hall, D. R., Xia, B., Porter, K. A., Padhorny, D., Yueh, C., Vajda, S. (2017). The ClusPro web server for protein–protein docking. *Nature protocols*, *12*(2), 255-278.

5. Seeliger, D.; De Groot, B.L. Ligand docking and binding site analysis with PyMOL and Autodock/Vina. J. Comput. Aided Mol. Des. 2010, 24, 417–422.

6. Bowers, K.J.; Chow, D.E.; Xu, H.; Dror, R.O.; Eastwood, M.P.; Gregersen, B.A.; Klepeis, J.L.; Kolossvary, I.; Moraes, M.A.; Sacerdoti, F.D. Scalable algorithms for molecular

dynamics simulations on commodity clusters. In Proceedings of the SC'06: 2006 ACM/IEEE Conference on Supercomputing, Tampa, FL, USA, 11–17 November 2006; p. 43.

7. Horner-Miller, B. CellSs: A programming model for the cell BE architecture. In Proceedings of the 2006 ACM/IEEE Conference on Supercomputing, Tampa, FL, USA, 11–17 November 2006; Association for Computing Machinery: New York, NY, USA, 2006.

8. Schrodinger Maestro; LLC: New York, NY, USA, 2009; Available online: https://www.schrodinger.com/products/maestro.

9. Jo, S.; Kim, T.; Iyer, V.G.; Im, W. CHARMM-GUI: A web-based graphical user interface for CHARMM. J. Comput. Chem. 2008, 29, 1859–1865.

10. Humphrey, W.; Dalke, A.; Schulten, K. VMD: Visual molecular dynamics. J. Mol. Graph. 1996, 14, 33–38

11. Kim, S.; Oshima, H.; Zhang, H.; Kern, N.R.; Re, S.; Lee, J.; Roux, B.; Sugita, Y.; Jiang, W.; Im, W. CHARMM-GUI Free Energy Calculator for Absolute and Relative Ligand Solvation and Binding Free Energy Simulations. J. Chem. Theory Comput. 2020, 16, 7207–7218.
